# Digital Spatial Profiling of Individual Glomeruli From Patients With Anti-Neutrophil Cytoplasmic Autoantibody-Associated Glomerulonephritis

**DOI:** 10.3389/fimmu.2022.831253

**Published:** 2022-03-02

**Authors:** Lin Ye, Yu Liu, Xuejing Zhu, Tongyue Duan, Chang Wang, Xiao Fu, Panai Song, Shuguang Yuan, Hong Liu, Lin Sun, Fuyou Liu, Kyung Lee, John Cijiang He, Anqun Chen

**Affiliations:** ^1^Department of Nephrology, Hunan Key Laboratory of Kidney Disease and Blood Purification, Institute of Nephrology, The Second Xiangya Hospital at Central South University, Changsha, China; ^2^Division of Nephrology, Department of Medicine, Icahn School of Medicine at Mount Sinai, New York, NY, United States; ^3^Renal Program, James J. Peters Veterans Affairs Medical Center at Bronx, New York, NY, United States

**Keywords:** digital spatial profiling (DSP), anti-neutrophil cytoplasmic antibody (ANCA), glomerulonephritis, Bowman’s capsule, complement, secreted phosphoprotein 1 (SPP1), CD44, fibrosis

## Abstract

We previously showed that the rupture of Bowman’s capsule (BC) promotes the progression of crescentic glomerulonephritis by enhancing the entry of CD8^+^ T cells into the glomeruli. In the present study, we utilized digital spatial profiling to simultaneously profile the altered abundances of the messenger RNA (mRNA) transcripts and proteins in the glomerular and periglomerular areas of four biopsy samples of anti-neutrophil cytoplasmic autoantibody-associated glomerulonephritis (ANCA-GN) and two biopsy specimens of minimal change disease (MCD) controls. The paraffin-embedded biopsy samples were stained with collagen IV, CD45, and SYTO 13 to distinguish the glomeruli with periglomerular infiltration but intact BC, with focal BC rupture, and with extensive rupture of BC and glomeruli without crescent formation and leukocytic infiltration in ANCA-GN. By assessing multiple discrete glomerular areas, we found that the transcript expression levels of the secreted phosphoprotein-1 and its receptor CD44 were upregulated significantly in the glomeruli with more severe ruptures of BC, and their expression levels correlated positively with the fibrotic markers. We also found that both alternative and classic complement pathways were activated in the glomeruli from patients with ANCA-GN. Furthermore, M1 macrophages were involved mostly in the early stage of BC rupture, while M2 macrophages were involved in the late stage and may contribute to the fibrosis process of the crescents. Finally, loss of glomerular cells in ANCA-GN was likely mediated by apoptosis. Our results show that digital spatial profiling allows the comparative analysis of the mRNA and protein profiles in individual glomeruli affected differently by the disease process and the identification of potential novel mechanisms in ANCA-GN.

**Graphical Abstract:**
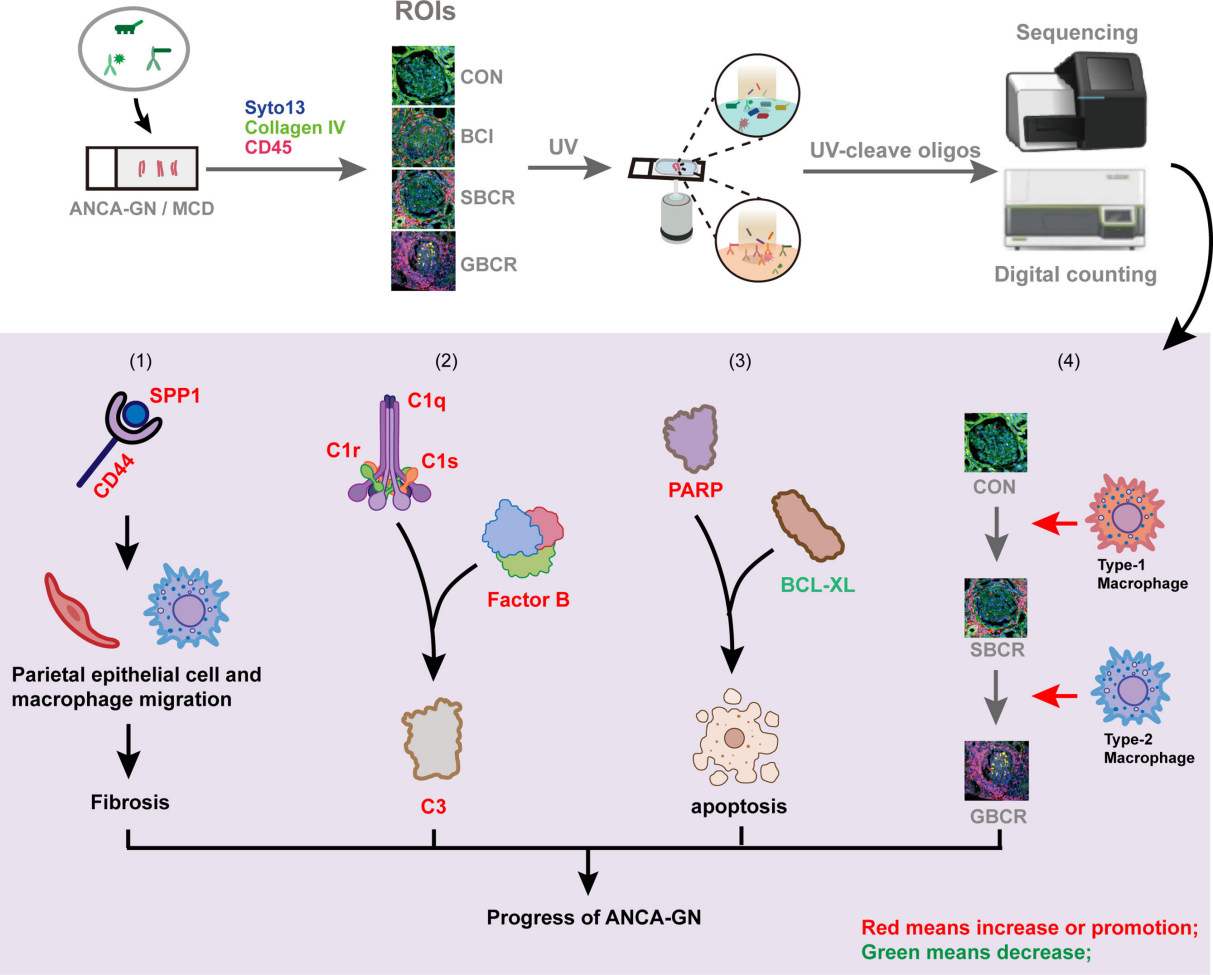


## Introduction

Anti-neutrophil cytoplasmic autoantibody-associated glomerulonephritis (ANCA-GN) is defined as a pauci-immune and necrotizing crescentic glomerulonephritis (GN) that is associated with the rapid loss of renal function and proteinuria, frequently progressing to end-stage kidney disease (1). The exact pathogenesis of this disease remains unclear. The glomerular injury in ANCA-GN involves the loss of podocytes, cells critical for maintaining glomerular architecture and function. Podocytes are located in a special environment that is separated from the outside tissues by the Bowman’s capsule (BC) and separated from the capillary lumen by the glomerular basement membrane (2).

We have recently shown that the BC provides a protective barrier by preventing the influx of cytotoxic CD8^+^ T cells into BC under normal physiological conditions (3). Even after the induction of mild nephrotoxic serum-induced nephritis, the CD8^+^ T cells directed at the podocyte-specific epitope [enhanced green fluorescent protein (EGFP)-specific Jedi T cells] accumulated around the injured glomeruli in mice, with podocyte-specific EGFP expression together with other inflammatory cells, but were not found within the Bowman’s space, provided that the BC was intact. However, in more severely damaged glomeruli with BC rupture, a massive influx of CD8^+^ T cells was observed within the Bowman’s space, with concomitant destruction of EGFP^+^ podocytes and a catastrophic rapidly progressive GN (3). Indeed, recent studies have shown that BC rupture is associated with severe deterioration of kidney function, evidenced by the higher cumulative incidence of renal replacement therapy within 30 days after admission than in those without BC rupture (4). Consistently, BC rupture can be used as a risk marker to improve the outcome prediction for ANCA-GN (5).

Based on these novel findings and previous reports (6), we hypothesized an integrated “two-stage” model of crescentic GN progression, where the initial insult to the glomerular capillaries in various forms of crescentic GN (first stage) results in podocyte injury through injured glomerular endothelial cell–podocyte crosstalk and/or through infiltrating leukocytes, leading to proteinuria. The release of neoepitopes from damaged podocytes (second stage) results in the activation of dendritic cells and in the subsequent activation and proliferation of CD4^+^ and CD8^+^ T cells specific for these epitopes. At the early stage when BC is intact, CD8^+^ T cells and other immune cells, activated by cytokines and chemokines released by the injured glomeruli, migrate and accumulate near and around the glomerulus. As the disease progresses, activated parietal epithelial cells and infiltrated periglomerular immune cells may secrete proteases causing BC rupture, allowing the entry of inflammatory immune cells, including CD8^+^ T cells, into the Bowman’s space to destroy the neoepitope-carrying podocytes and further damage the glomeruli (7).

To assess the difference between glomeruli with or without BC rupture, we used digital spatial profiling (DSP), a novel technology capable of medium- to high-plex spatial profiling of proteins and RNAs in formalin-fixed paraffin-embedded (FFPE) section samples (8). One of the advantages of DSP compared to other spatial transcriptome technologies is its ability to sectionalize specific regions of interest (ROIs) with high magnification, defined by the researchers, using multiple fluorescence labeling, thereby enabling the analysis of multiple proteins and RNAs (9). Using this novel method, we performed a “pilot study” with kidney sections from four patients with ANCA-GN and two minimal change disease (MCD) controls. We especially selected the areas with or without BC rupture accompanied by periglomerular infiltration in the kidneys of ANCA-GN patients in order to profile the expressions of the RNAs and proteins specifically in these areas.

A comparison of the expression profiles of the glomeruli affected differently by the disease process provided new insights into the mechanisms of ANCA-GN progression.

## Results

### Digital Spatial Profiling of Peri-Glomerular and Intra-Glomerular Areas of Renal Biopsy Samples With ANCA-GN

**Figure 1:**
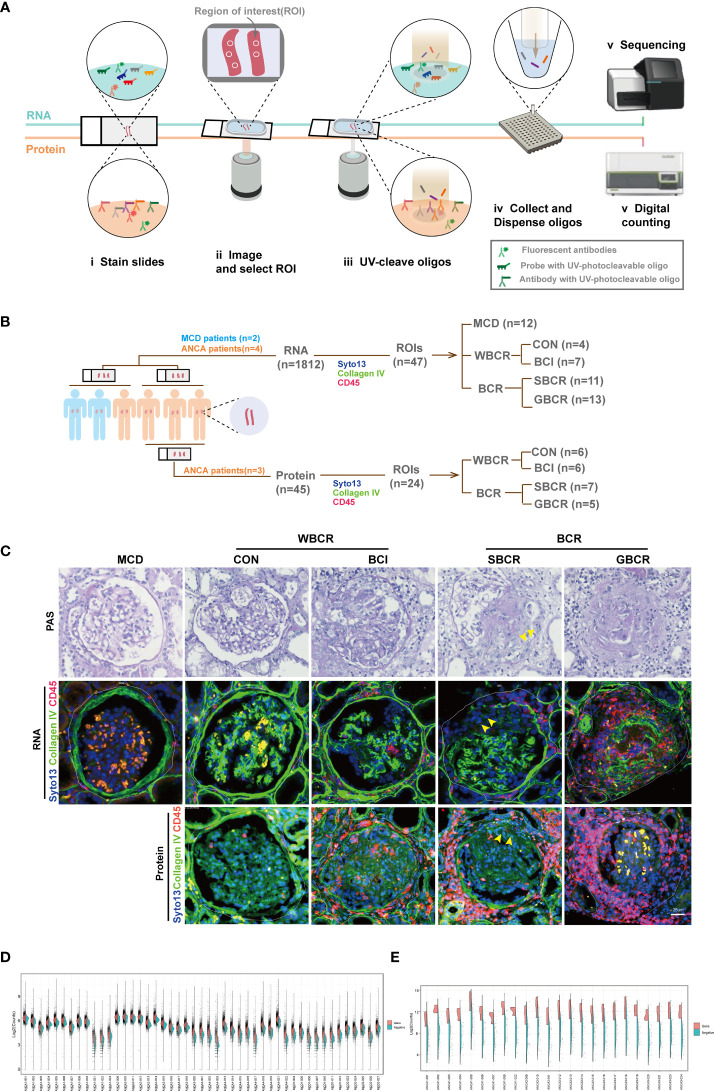
Digital spatial profiling of renal biopsies from patients with ANCA-GN and MCD. **(A)** Schematic overview of the DSP workflow. **(B)** Information on the glomeruli included in this analysis. Four patients with ANCA-GN and two patients with MCD were enrolled. The region of interest (ROI) was selected evenly from patients according to the immunofluorescence staining with collagen IV, CD45, and SYTO 13. For the RNA profiles, the glomeruli from MCD (*n* = 12), from ANCA-GN with crescent formation but intact BC (BCI, *n* = 7), with segmental BC rupture (SBCR, *n* = 11), with global BC rupture (GBCR, *n* = 13), and without crescent formation and leukocyte infiltration (CON, *n* = 4) were included for analysis. For the protein profiles, glomeruli with the characteristics of CON (*n* = 6), BCI (*n* = 7), SBCR (*n* = 5), and BCR (*n* = 7) were included for further analysis. **(C)** Representative images from each group. *Scale bar*, 25 μm. **(D)** Split violin plot of the RNA counts by ROIs. Gene counts are shown on the *left* (*orange*) and the negative probe counts are displayed on the *right* (*green*). **(E)** Split violin plot of the protein counts by ROIs. *ANCA-GN*, anti-neutrophil cytoplasmic autoantibody-associated glomerulonephritis; *MCD*, minimal change disease; *DSP*, digital spatial profiling; *WBCR*, without Bowman’s capsule rupture; *BCR*, Bowman’s capsule rupture; *BC*, Bowman’s capsule.

To explore the mechanisms of BC rupture on the progression of ANCA-GN, the GeoMx^®^ Digital Spatial Profiler was used to quantitate the expressions of specific panels of genes and proteins in the spatially defined regions. Together with the fluorescent-labeled antibodies, ultraviolet (UV)-photocleavable oligos (DSP barcodes) linked with target complementary sequences (RNA) or target antibodies (protein) were co-incubated. After the ROIs were selected, the specific regions were lit up with UV. Subsequently, the DSP barcodes were collected and amplified for further Illumina sequencing (RNA) or linked with a fluorescent label reporter for further counting with the NanoString counter machine (protein) (**Figure 1A** and **Supplementary Figure S1**).

The continuity of collagen IV-positive staining represented the integrity of the BC, CD45-positive staining showed the infiltration of immune cells, while SYTO 13 staining was used to label the cell nuclei. The glomeruli were selected evenly from two renal biopsies with MCD (*n* = 12). The following groups were included for transcription profiling: four ANCA-GN renal biopsies with normal appearance [control (CON), *n* = 4], periglomerular infiltration but with intact BC (BCI, *n* = 7), segmental BC rupture (SBCR, *n* = 11), and global BC rupture (GBCR, *n* = 13) (**Figure 1B**). For the protein assay, because the panel of proteins mainly focused on immune-related cells and the two renal samples from patients with MCD were patched with a renal sample from the fourth patient with ANCA, we only selected the renal samples from three other ANCA patients for protein analysis owing to cost considerations. Glomeruli with the characteristics of CON (*n* = 6), BCI (*n* = 7), SBCR (*n* = 5), and GBCR (*n* = 7) were selected from three ANCA-GN renal biopsies and were included in the protein assay after passing quality control (**Figure 1B**).

For some analyses, the CON and BCI groups were combined into WBCR (without BC rupture) and the SBCR and GBCR groups combined into BCR (BC rupture). Representative pictures are shown in **Figure 1C**. The demographic and clinical features of each patient are displayed in **Supplementary Table S1**, and detailed information on the histopathology scoring of the ANCA-GN patients is shown in **Supplementary Table S2**.

For the RNA DSP assay, the probes specific for 1,833 genes are listed in detail in **Supplementary Table S2**. The geometric mean of the housekeeping genes was calculated logarithmically (log2), and the histogram of the mean per ROI is displayed in **Supplementary Figure S2A**. The surface area and nuclei counts of all ROIs passed quality control (**Supplementary Figure S2B**). The housekeeping gene *TMUB2* correlated well with the other housekeeping genes such as *ARMH3* and *TLK2* (**Supplementary Figure S2C**). Normalization with a housekeeper and the Q3 method matched very well (**Supplementary Figure S2D**); therefore, the Q3 method was used for normalization, as recommended. The count distributions of the genes and negative controls after normalization from each ROI are displayed in **Figure 1D**.

For the protein DSP assay, the probes targeting 45 proteins are provided in detail in **Supplementary Table S3**. The geometric mean of the housekeeping proteins was calculated logarithmically (log2), and the histogram of the mean per ROI is displayed in **Supplementary Figure S3A**. The surface area and nuclei counts of all ROIs passed quality control (**Supplementary Figure S3B**). The ratio of the count value of each target in each ROI to the count value of immunoglobulin G (IgG) was calculated logarithmically (log2), and the proteins with log values <0 (labeled blue), including CD80, FOXP3, CD66b, PD-L1, PD-L2, and CD95/Fas, were excluded from further analysis (**Supplementary Figure S3C**). Housekeeping proteins were used to normalize the raw protein counts (**Supplementary Figure S3D**). The count distribution of the proteins after normalization from each ROI are shown in **Figure 1E**.

### Involvement of the Classic Complement Pathway in the Pathogenesis of ANCA-GN

**Figure 2:**
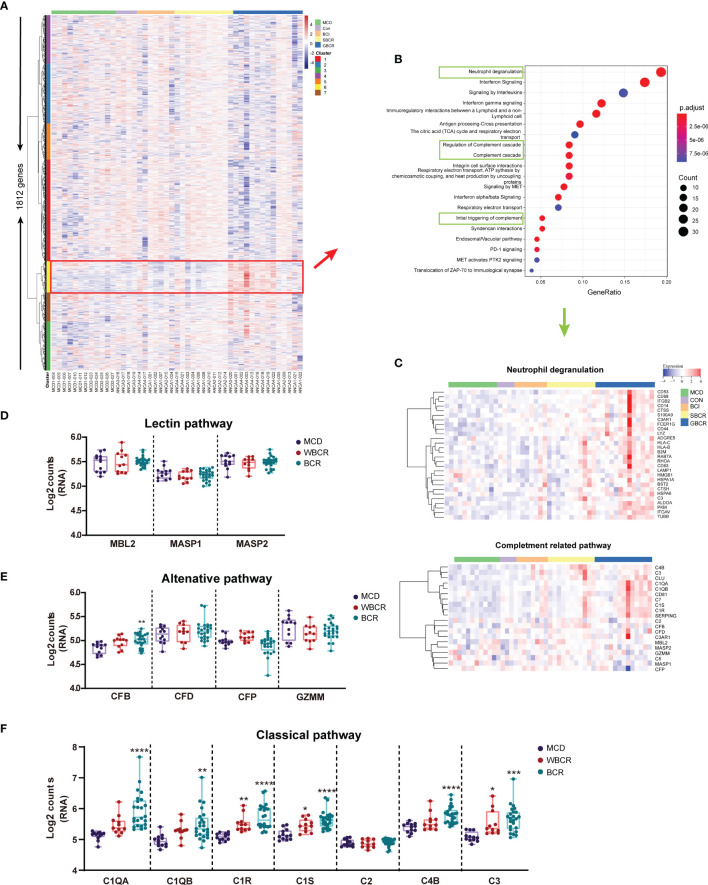
The classic complement pathway is involved in the pathogenesis of anti-neutrophil cytoplasmic autoantibody-associated glomerulonephritis (ANCA-GN). **(A)** Heatmap of the 1,813 detected genes. Cluster analysis indicated seven main clusters marked in *different colors* (*left*). **(B)** Pathway enrichment analysis of cluster 6 [*red box* in **(A)**]. **(C)** Neutrophil degranulation and complement-related pathways are highlighted in *green box*, and the heatmap of related genes is displayed separately. **(D)** RNA expression of the complement pathway of lectin-related genes. **(E)** RNA expression of the alternative complement pathway-related genes. **(F)** RNA expression of the classic complement pathway-related genes. All data were analyzed by one-way analysis of variance (ANOVA). Compared with the minimal change disease (MCD) group: *p < 0.05, **p < 0.01, ***p < 0.001, and ****p < 0.0001.

The heatmap of the normalized counts of each ROI is displayed in **Figure 2A**. The genes of cluster 6 were highlighted in the red box, the pathway enrichment analysis of which is provided in **Figure 2B**. The neutrophil degranulation and complement-related pathways, including initial triggering of complement, regulation of complement cascade, and complement cascade, were significantly enriched (**Figure 2B**). The genes related to neutrophil degranulation and complement-related pathways are listed separately in **Figure 2C**, with the majority of genes involved in neutrophil degranulation found to be upregulated. The genes involved in the complement pathway are displayed separately in **Figures 2D**–**F**. There was no significant difference with regard to the genes involved in the lectin pathway, including mannose-binding lectin 2 (*MBL2*), MBL-associated serine protease 1 (*MASP1*), and MBL-associated serine protease 2 (*MASP2*) (**Figure 2D**). Complement factor B (CFB) increased significantly with the rupture of BC, but the other genes involved in the alternative complement pathway, including complement factor D (*CFD*), complement factor properdin (*CFP*), and granzyme M (*GZMM*), showed no difference (**Figure 2E**). Notably, most of the genes involved in the classic complement pathway were remarkably elevated in the group with BC rupture compared with the MCD group. These genes included complement C1q A chain (*C1QA*), complement C1q B chain (*C1QB*), complement C1r (*C1R*), complement C1s (*C1S*), complement C4b (*C4B*), and complement C3 (**Figure 2F**). Especially, the expression levels of *C1R* and *C1S* markedly increased in the glomeruli from ANCA-GN patients even without rupture of the BC compared with the MCD group. These results showed that the classic complement pathway is likely activated in ANCA-GN.

### *SPP1* Signaling Contributes to the Fibrosis Pathway in ANCA-GN

**Figure 3:**
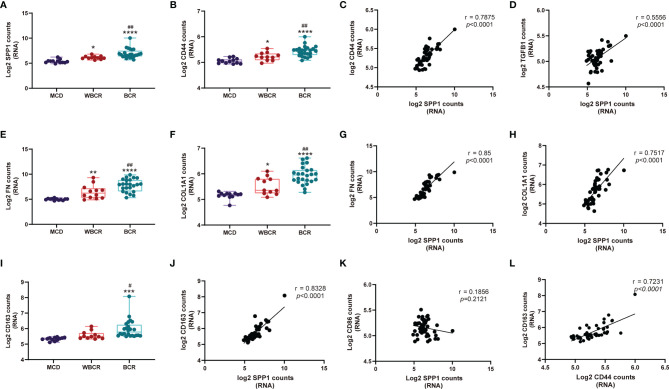
SPP1 signaling contributes to the fibrosis pathway in anti-neutrophil cytoplasmic autoantibody-associated glomerulonephritis (ANCA-GN). **(A, B, E, F, I)** RNA expression of *SPP1*, *CD44*, *COL1A1*, *FN*, and *CD163* with worsening of glomerular injury. **(C, D, G, H, J, K)** Correlation of the RNA expressions between *SPP1* and *CD44*, *TGF-β1*, *COL1A1*, *FN*, *CD163*, and *CD86*. **(L)** Correlation of the RNA expressions between *CD44* and *CD163*. All data were analyzed by one-way analysis of variance (ANOVA). Compared with the MCD (minimal change disease) group: *p < 0.05, **p < 0.01, ***p < 0.001, and ****p < 0.0001. Compared with the WBCR (without Bowman’s capsule rupture) group: ^#^*p* < 0.05 and ^##^*p* < 0.01. *SPP1*, secreted phosphoprotein 1; *COL1A1*, collagen type I alpha 1 chain; *FN*, fibronectin 1.

Among the 1,812 genes detected, secreted phosphoprotein 1 (*SPP1*), also named osteopontin (OPN), was remarkably increased in the WBCR and BCR groups compared to the MCD group. Its transcription level notably increased in the BCR group compared to that in the WBCR group, indicating its pathologic role in the progression of ANCA-GN (**Figure 3A**). CD44 functioned as the receptor of SPP1 (10), which showed a similar expression pattern to *SPP1* (**Figure 3B**), and their expressions showed a strong positive correlation (*r* = 0.7875, *p* < 0.0001) (**Figure 3C**). *SPP1* is considered a major driver for renal fibrosis (11), the secretion of which is stimulated by inflammatory cytokines (12) and the suppression of which could alleviate kidney fibrosis (13). In our study, the expressions of fibronectin and Col1A1 were gradually elevated in the WBCR and BCR groups compared to those in MCD (**Figures 3E, F**). Their expressions were strongly positively correlated with SPP1 (*r* = 0.85, *p* < 0.0001; *r* = 0.7517, *p* < 0.0001, respectively) (**Figures 3G, H**). Consistently, SPP1 was also moderately positively correlated with TGF-b1, a master regulator of fibrosis (*r* = 0.5556, *p* < 0.0001) (**Figure 3D**).

The above results highlighted the critical role of *SPP1* in glomerulosclerosis and possibly in fibrous crescent formation. Moreover, *SPP1* is known to mediate M2-type macrophage polarization (14), which also plays a role in fibrosis (15, 16). Our data showed that the expression of CD163 gradually increased with worsening of the glomerular injury (**Figure 3I**), and SPP1, together with its receptor CD44, strongly correlated with CD163 (an M2-type macrophage marker; *r* = 0.8328, *p* < 0.0001; *r* = 0.7231, *p* < 0.0001, respectively) (**Figures 3J, L**), but not with CD86 expression (an M1-type macrophage marker; *r* = 0.1856, *p* = 0.2121) (**Figure 3K**).

### Protein Profiling Reveals That Apoptosis Is the Main Pathway Responsible for Glomerular Cell Injury in ANCA-GN

**Figure 4:**
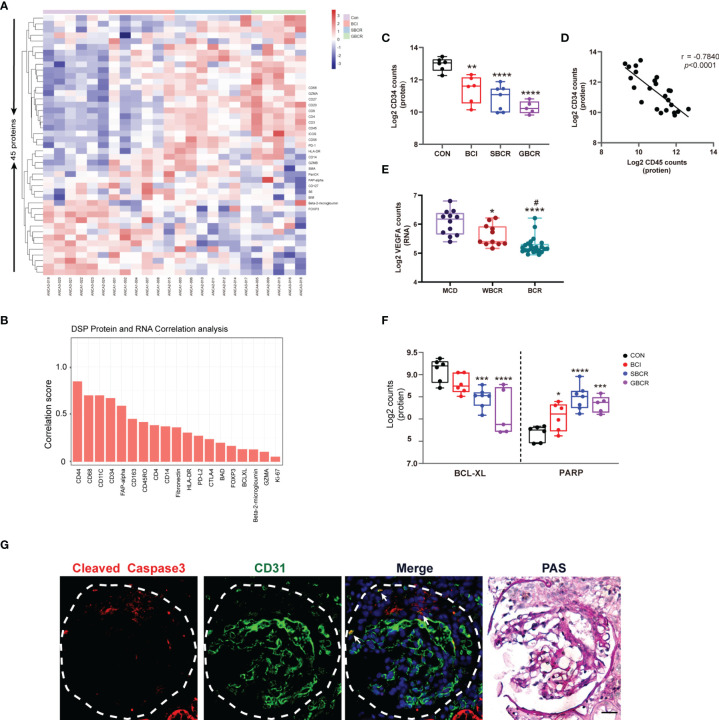
Protein profiles indicating that apoptosis might be the main pathway responsible for the glomerular cell injury in anti-neutrophil cytoplasmic autoantibody-associated glomerulonephritis (ANCA-GN). **(A)** Heatmap of 45 detected proteins. **(B)** Correlation analysis of common proteins and RNAs of individual targets generated from digital spatial profiling (DSP) based on all regions of interest (ROIs). **(C)** Protein expression of CD34 with worsening of the glomerular injury. **(D)** Protein expression correlation of CD34 and CD45. **(E)** Transcription expression of VEGFA with worsening of the glomerular injury. **(F)** Protein expressions of BCL-XL and PARP. **(G)** Immunofluorescence co-staining of caspase-3 and CD31 in serial renal biopsy specimens of patients with ANCA-GN. *Scale bar*, 25 μm. The image of collagen IV staining was from the serial kidney section for protein DSP. PAS staining was performed on the same slide after co-staining with caspase-3 and CD31. Compared with the CON (control) group: *p < 0.05, **p < 0.01, ***p < 0.001, and ****p < 0.0001. Compared with the WBCR (without Bowman’s capsule rupture) group: ^#^*p* < 0.05. *VEGFA*, vascular endothelial growth factor A; *BCL-XL*, B-cell lymphoma—extra large; *PARP*: poly(ADP-ribose) polymerase; *PAS*, periodic acid–Schiff.

To confirm the finding in the DSP RNA profile, the immune-related proteins were detected in ANCA-GN applying DSP protein analysis. Forty-five proteins, including three housekeeping proteins, were detected together with three negative controls. Their expression heatmap is displayed in **Figure 4A**. Genes such as *CD44*, *CD68*, *CD11C*, *CD34*, and *FAP* exhibited strong correlations at the RNA and protein levels, whereas *BAD*, *FOXP3*, *BCLXL*, and *B2M* exhibited weak correlations (**Figure 4B**). CD34, an endothelial cell marker, was remarkably decreased in the BCI, SBCR, and GBCR groups compared with the CON group. Their expression levels also showed a gradual decrease with more severe rupture of the BC and worsening of the glomerular injury (**Figure 4C**). Furthermore, the CD34 protein level was strongly negatively correlated with CD45 (*r* = −0.7840, *p* < 0.0001), suggesting a close relationship between leukocyte infiltration and endothelium injury in ANCA-GN (**Figure 4D**). Consistently, vascular endothelial growth factor A (VEGFA), produced by podocytes, also decreased progressively with worsening of glomerular injury (**Figure 4E**). In addition, the expression of BCL-XL, an anti-apoptotic protein, was decreased significantly in the BCI, SBCR, and GBCR groups compared with that in the CON group (**Figure 4F**). The expression of PARP, a protein involved in programmed cell death, was significantly increased in the BCI, SBCR, and GBCR groups compared with that in the CON group (**Figure 4F**). CD34 is a cell surface transmembrane protein that is often shed from endothelial cells when they are injured. To determine whether apoptosis contributes to the injury of endothelial cells, we performed immunofluorescence co-staining of caspase-3 and CD34. As shown in **Figure 4G**, caspase-3 staining was co-localized with CD34. These results imply that apoptosis might be responsible for glomerular cell injury in ANCA-GN.

### DSP Identified That the Types of Immune Cell Infiltration Were Different During BC Rupture

**Figure 5:**
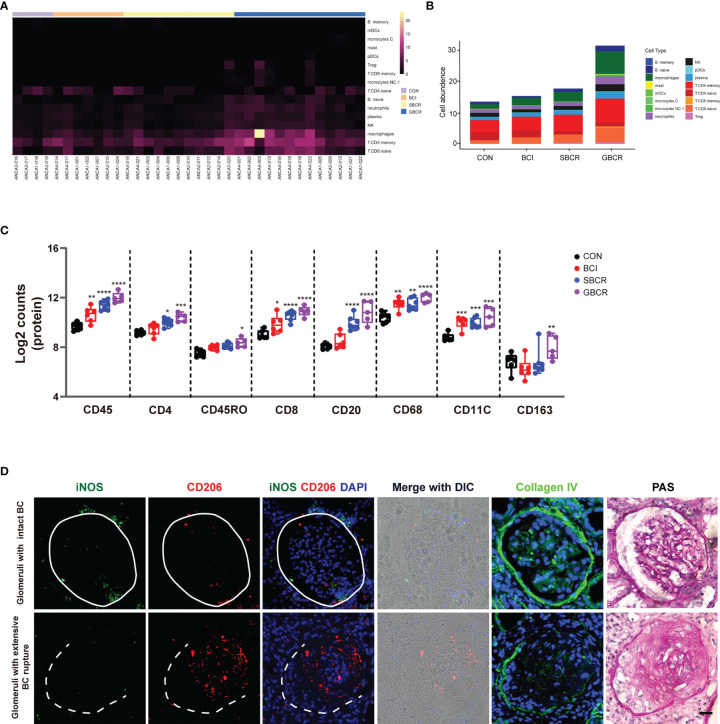
Different types of immune cell infiltration during rupture of the Bowman’s capsule. **(A)** Heatmap of the cell abundance of immune cell infiltration based on the regions of interest (ROIs) selected from anti-neutrophil cytoplasmic autoantibody-associated glomerulonephritis (ANCA-GN) patients. **(B)** Average cell abundance of each category of immune cells in different groups. **(C)** Protein expressions of immune cell markers, including CD45, CD4, CD45RO, CD8, CD20, CD68, CD11C, and CD163. **(D)** Immunofluorescence co-staining of iNOS and CD206 in serial renal biopsy specimens of patients with ANCA-GN. *Scale bar*, 25 μm. Intact Bowman’s capsule is indicated by *solid circle*, while extensive Bowman’s capsule rupture is indicated by the *dotted line*. The image of collagen IV staining from the serial section for protein digital spatial profiling (DSP) indicated the integrity of the Bowman’s capsule. Periodic acid–Schiff (PAS) staining was performed on the same slide after co-staining with iNOS and CD206. Compared with the CON (control) group: *p < 0.05, **p < 0.01, ***p < 0.001, and ****p < 0.0001.

We investigated what cell types might be responsible for BC rupture. The 16 categories of immune cells generated from the transcription profiles of ROIs selected from ANCA-GN renal samples are displayed in **Figure 5A**, which showed more immune cell infiltration in the groups with BC rupture. The highest number of immune cells was observed in the GBCR group. When the data were rearranged into a bar chart, we found that the infiltration of memory CD4^+^ T cells, naive CD8^+^ T cells, memory CD8^+^ T cells, naive B cells, memory B cells, and macrophages increased, whereas the infiltration of naive CD4^+^ T cells decreased with more BC rupture and worse glomerular injury (**Figure 5B**). The protein profiles further confirmed the findings in the RNA analysis, which showed that CD8 and CD68 might be involved in the early development of BC rupture, whereas CD20 and CD4 might be involved in the late stage of progression (**Figure 5C**). When we classified CD68 macrophages into subgroups of M1 macrophages (CD11c) and M2 macrophages (CD163), the protein profiles indicated that M1 macrophages were involved mostly in the early stage of BC rupture, while M2 macrophages were involved in the late stage (**Figure 5C**). These findings were further confirmed by immunofluorescence staining with inducible nitric oxide synthase (iNOS) (another M1 macrophage marker) and CD206 (another M2 macrophage marker) (**Figure 5D**).

## Discussion

The renal damage secondary to ANCA-associated vasculitis appeared heterogeneous, and individual glomeruli showed different extents of injury, including crescent formation, infiltration of immune cells, and rupture of BC. In the same kidney, some glomeruli appeared quite normal, some glomeruli showed crescent formation but with intact BC, some glomeruli had focal BC rupture, and some glomeruli had extensive BC rupture with massive infiltration of immune cells. As we previously demonstrated, the BC can protect podocytes from damage, and its rupture promotes more infiltration of immune cells into the glomeruli, leading to more podocyte loss and rapid progression of GN (3). To understand the mechanisms of injury in these individual glomeruli with different degrees of injury, we profiled the transcriptomics and proteomics in different areas of ANCA-GN kidney by selecting those individual glomeruli with different degrees of BC rupture in FFPE samples using the DSP method. This study helped us to identify the underlying pathogenesis mediating the progression of ANCA-GN.

Although ANCA-GN was previously considered a pauci-immune necrotizing crescentic GN, accumulating evidence has shown that immune complex and complement deposition were present in the glomeruli of patients with ANCA-GN, which was associated with more severe proteinuria, higher percentages of crescents, and poorer renal function (1). Local complement dysregulation was identified as an essential component of crescent formation and disease progression (17). A recent study has shown that the C5a receptor inhibitor had renal protective effects in these patients (18). Consistent with this, our study suggests that multiple complement-related pathways, such as initial triggering of complement, regulation of complement cascade, and complement cascade, were enriched in the glomeruli from ANCA-GN.

Previous data reported that a greater degree of neutrophil degranulation could result in a more abundant local complement activation and account for the more severe renal damage in patients with ANCA-GN (19). Consistent with this, our study showed that, with worsening of the glomerular injury, the neutrophil degranulation pathway was significantly upregulated together with complement activation. The alternative complement pathway was thought to be critical in the pathogenesis of ANCA-GN for several reasons: 1) depletion of factor B, critical for the alternative complement pathway, could protect mice from this disease (20); 2) the membrane attack complex (MAC), C3d, factor B, and factor P were detected in renal biopsy samples with pauci-immune myeloperoxidase (MPO)-ANCA-GN (21); and 3) both the renal staining and the urinary and plasma levels of the proteins involved in the alternative complement pathway were significantly upregulated in ANCA-GN (20).

The common path of the classical and alternative complement pathways is the conversion of C3 to its active forms, C3a and C3b, the latter forming C5 convertase and subsequently converting C5 to C5a and C5b (1). C5a could recruit inflammatory cells such as neutrophils, monocytes, and T cells to the activation site, whereas C5b forms the MAC (1). In our experiment, we found that the RNA expression levels of C3 and CFB, together with C1QA, C1QB, and C1R, increased progressively with worsening of glomerular injury and more severe rupture of the BC, indicating that the classic complement pathway is also involved in the progression of glomerular injury in ANCA-GN. This was supported by the previous observation from others showing that C4d was found positive in the kidney in 70.8% of 187 renal biopsies in patients with ANCA-associated vasculitis (22). Previous studies suggested that activation of the complement was different between ANCA-positive and ANCA-negative GN (19). The study showed larger proportions of C3, C9, complement factor H-related protein 1, C4, C5, and immunoglobulins in ANCA-negative GN than in ANCA-positive GN. Previous studies were mostly obtained from immunofluorescence staining or mass spectrometry analysis of whole kidney biopsies, not considering the heterogeneous lesion of the individual glomeruli, which could mask the contribution of the classical complement pathway in disease progression. The reason for the activation of the classical complement pathway without visible electron-dense deposition might be the rapid degradation of local immune complexes in ANCA-GN (22).

SPP1, also named OPN, is a secreted protein mainly expressed in bone and epithelial tissues (10). It is present in healthy kidneys and other cell types, including activated T cells and macrophages, among others, and its expression is upregulated in the diseased condition (10). In crescentric GN, the mRNA and protein expressions of SPP1 were markedly upregulated (10). CD44 functions as the receptor of SPP1 (23), which is expressed on activated parietal epithelial cells (PECs) (24). The RNA expression level of SPP1 showed a strong positive correlation with that of CD44, and CD44 was identified to be regulated by phosphorylated extracellular signal-related kinase (pERK). CD44 upregulation was accompanied by a notably increased expression of collagen IV and the migration of parietal epithelial cells (25). Additionally, SPP1 is considered an epithelial–mesenchymal transition hallmark marker (15). Consistent with this, our data showed that the expression of SPP1 increased progressively with worsening of glomerular injury and was correlated with the expressions of fibronectin and collagen. Collectively, these findings suggest that SPP1 may promote the development of fibrous crescent by binding to CD44, thereby playing a pivotal role in the progression of ANCA-GN. Therefore, treatment specifically targeting the SPP1–CD44 axis can be considered for patients with ANCA-GN.

Our previous study showed that, with the rupture of BC, large proportions of CD8 and CD68 infiltrate the capillary loop (3). In this study, we found that CD8 and CD68 might be involved in the early development of BC rupture, whereas CD20 and CD4 might be involved during the late stage of progression. CD68 is widely accepted as a pan-macrophage marker, CD86 is known as a specific marker for M1 macrophages, and CD163 is considered as a marker for M2 macrophages (26). These data indicate that M1 macrophages might play a role in BC rupture, whereas M2 macrophages increase at the late stage of the disease, which might contribute to fibrosis. In addition, we found that the RNA and protein expression levels of CD163 increased significantly when the BC ruptured, together with the increased expression of SPP1, which was reported to be significantly correlated with macrophage infiltration and M2 polarization (15). It was previously reported that both the high expression of SPP1 and that of its receptor CD44 correlated with an increased macrophagic infiltration (27). Together, our data suggest that early M1 macrophage infiltration may be involved in the rupture of BC and that SPP1-mediated M2 macrophage polarization may contribute to glomerulosclerosis and crescent fibrosis.

Our previous data showed that, upon BC rupture, a greater number of podocytes were lost (3). Our present study shows that with the progression of the disease, the apoptotic marker PARP was markedly increased, whereas the anti-apoptosis protein BCL-XL decreased significantly, which may be due to the increased podocyte apoptosis. In addition, the expression of VEGFA notably decreased with the progression of individual glomeruli, consistent with podocyte loss. CD34, a marker of endothelial cells, decreased drastically with worsening of the glomerular lesion, indicating the loss or injury of the glomerular endothelial cells in ANCA-GN. Interestingly, the protein expression level of CD34 showed a negative correlation with that of CD45, a marker of leukocytes, indicating that endothelial cell injury is tightly associated with the infiltration of immune cells in the glomeruli. Taken together, these data imply that apoptosis might be one of the mechanisms responsible for the loss of podocytes and glomerular endothelial cells in ANCA-GN, which is consistent with a previous study indicating that apoptotic regulation is important in the development of pathologic glomerular sclerosis in crescentic GN (3, 28).

This study had some limitations. Firstly, the sample size was not large enough due to cost limitations and therefore may not detect all the variations in patients with ANCA-GN. Secondly, the RNA target panel included only 1,833 genes and the protein analysis included only 45 proteins. Wider and deeper gene/protein panels will provide more comprehensive information. Thirdly, the preliminary results needed to be validated by other methods. Therefore, the mechanism underlying the results needs to be investigated further.

In conclusion, our study applied novel special transcriptomic and proteomic technology to profile the gene and protein expressions in individual glomeruli with different degrees of BC rupture. Our study demonstrated that both classic and alternative complement pathways are involved in the progression of ANCA-GN, that the SPP1–CD44 axis plays a dominant role in the development of fibrous crescent, and that apoptosis might be the important mechanism leading to the loss of glomerular cells in ANCA-GN.

## Materials and Methods

### Study Population and Ethical Approval

FFPE tissues were obtained from patients who underwent renal biopsy and were diagnosed with ANCA-GN (*n* = 4) and MCD (*n* = 2) from July 2020 to July 2021. Patients’ clinical information is provided in **Supplementary Table S1**. This study was approved by the Ethics Committee of the Second Xiangya Hospital of Central South University (approval no. 2021-072). Written informed consent for tissue use in research was obtained at the Second Xiangya Hospital of Central South University. The renal biopsy sections measuring 5 µm in thickness from three patients with ANCA-GN were patched into one DSP slide, and those from other patients with ANCA-GN together with those from two patients with MCD were patched into another DSP slide. The histopathology scores generated specifically from the serial kidney sections for the DSP profiles were evaluated independently by two pathologists according to previous literature (29). Differences in the scoring between the two pathologists were resolved by reviewing the biopsies to reach a consensus.

### General Description of DSP

DSP was performed according to the literature (8, 9, 30). Briefly, DSP barcodes containing UV-photocleavable oligos comprising collagen IV (a marker for BC integrity), CD45 (a marker for immune cells), and SYTO 13 (nuclei) to allow for the morphological outline of interesting areas were used synchronously. The DSP slides were stained with immunofluorescent antibodies to identify the glomerulus and the BC, followed by high-resolution scanning (×20 objective lens) using the DSP instrument to allow ultimate precise selection for ROIs. Different shapes of regions, such as geometric, segment, contour, gridded, and any polygon shapes, were accepted for ROI selection. The DSP barcodes of RNAs linked with UV-photocleavable oligos targeted the complementary sequences of specific mRNAs. Similarly, the DSP barcodes of proteins linked with UV-photocleavable oligos targeted specific proteins (antigens). The barcodes were released by UV light shed on the selected ROIs in the micro-sized programmable digital micromirror device (DMD; NanoString Technologies, Inc. Seattle, WA, USA), which were collected subsequently into a 96-well plate. Each barcode had its own unique sequence to distinguish from one another. The DSP barcodes collected from the RNA assay procedure were amplified by PCR, and subsequent sequencing was performed using the Illumina sequencing platform. Concurrently, the DSP barcodes generated from the protein probes were collected and hybridized with fluorescent label reporters for further fluorescence counting (proteins).

### DSP of RNA

Sections were deparaffinized in sequential xylene and rehydrated in graded ethanol. Target retrieval was performed in a water bath with 1 M Tris-EDTA buffer (pH 9.0) for 15 min. The FFPE samples were stained with a primary mouse CD45 antibody (13917; Cell Signaling Technology, Danvers, MA, USA) and subsequently with the Alexa Fluor 594 secondary mouse antibody (A-11012; Thermo Scientific, Waltham, MA, USA), Alexa Fluor 555-labeled collagen IV antibody, and the DNA stain SYTO 13 (GMX-PRO-MORPH-HST-12; NanoString Technologies Inc.) to identify the tissue morphology. The Alexa Fluor 555-labeled collagen IV antibody was covalently labeled using the collagen IV antibody (ab6586; Abcam, Cambridge, UK) with Alexa Fluor 555 Conjugation Kit—Lightning-Link (ab269820; Abcam) according to the protocol, and the resulting final concentration was 0.5 mg/ml. The slides were incubated with the aforementioned fluorescent antibodies in addition to the RNA probe sets (1,833 genes with 8,659 pairs of probes, provided with detail in **Supplementary Table S2**), which is similar to conventional immunofluorescence or immunohistochemistry (IHC). Stained slides were scanned with the GeoMx™ Digital Spatial Profiler (NanoString Technologies, Inc.). The ROIs were selected according to the integrity of the BC outlined by collagen IV staining, the leukocyte infiltration marked by CD45 staining, and crescent formation indicated by the large numbers of nuclei labeled by SYTO 13. Glomeruli with crescent formation but intact BC (BCI; *n* = 7 ROIs), with segmental or focal BC rupture (SBCR; *n* = 11 ROIs), with global or extensive BC rupture (GBCR; *n* = 13 ROIs), and glomeruli without crescent formation and leukocyte infiltration (control, CON; *n* = 4 ROIs) selected from four renal biopsy specimens from ANCA-GN patients and from two renal biopsy specimens from MCD patients (*n* = 12 ROIs) were included for further study. The selection of ROIs was performed by two professional pathologists to estimate the crescent formation, infiltration of leukocytes, and the degree of BC rupture.

### DSP of Protein

After deparaffinization and rehydration, antigen retrieved from renal slides was soaked in 1× citrate buffer, pH 6.0 (C9999; Sigma, St. Louis, MO, USA), in a preheated pressure cooker for 15 min. Then, the slides were stained with a Texas red-labeled CD45 antibody (GMX-PRO-MORPH-HST-12; NanoString Technologies, Inc.), Alexa Fluor 555-labeled collagen IV, and SYTO 13, together with the DSP antibody barcodes including immune cell core profiling, the immune activation status module, immune cell typing module, and cell death module, which are provided in detail in **Supplementary Table S3**. The corresponding ROIs from the RNA slides were selected for protein detection, whenever possible. The photocleaved oligos from all of the ROIs were collected using a similar method to the RNA profiling, then reacted directly with NanoString’s probe R and probe U (GMX-PRO-MORPH-HST-12 and GMX-PRO-HYB-96; NanoString Technologies, Inc.), which carried the fluorescence reporters and can be used for fluorescence counting with the nCounter^®^ MAX Analysis System (NanoString Technologies, Inc.).

### Library Preparation and RNA Sequencing

The UV-cleaved oligos from each ROI were collected, followed by PCR amplification using a pair of primers with the following sequences: forward: CAAGCAGAAGAC GGCATACGAGATXXXXXXXXGTGACTGGAGTTCAGACGTGTGCTCTTCCGATCT; reverse: AATGATACGGCGACCACCGAGATCTACAC XXXXXXXXACACTCTTTCCCTACACGACGCTCTTCCGATCT (GeoMx Seq Code Pack, GMX-NGS-SEQ-AB; NanoString Technologies, Inc.). ROI identity was preserved in the custom Illumina i5/i7 unique dual indexing sequences of the primer pairs (marked as X). The PCR products were amalgamated, mixed with AMPure XP beads (A63881; Beckman Coulter, Brea, CA, USA) twice for purification, and then sequenced. A high-sensitivity DNA Bioanalyzer chip (C105102-S1; BiOptic, New Taipei City, Taiwan) was used to measure the concentration and purity of the library. The Illumina NovaSeq instrument (Illumina, Inc., San Diego, CA, USA) was applied for sequencing paired ends (2 × 27-bp reads).

### Data Processing and Analysis

Reads were aligned to reveal the unique identities of the probe after sequencing. PCR duplicate reads were removed according to the unique identifier regions, followed by converting reads to digital counts. RNA sequencing saturation was set at 50%, as recommended by NanoString. The final count value of each gene was the arithmetic average of the rest of the individual probe counts after eliminating the outlier probes. The limit of quantitation (LOQ) was calculated based on the distributions of the negative control probes and was intended to approximate the quantifiable limits of gene expression per segment. The formula used to calculate the LOQ in an *i*-th segment was LOQ*_i_* = geomean (NegProbei) × geoSD (NegProbei)*n*. Two geometric standard deviations above the geometric means were typically used as the LOQ. Of the 1,833 detected genes, 660 (36%) were above the LOQ in at least one ROI, and all the genes, except the 21 negative genes, were included for further analysis. With regard to normalization, the methods used included Q3 (top 25%), based on the genes whose expression levels were in the top 25%, housekeeping, and negative probe. If the correlations among the three methods are good, either of them can be chosen; otherwise, the Q3 method is recommended. In our experiment, the Q3 method was used for normalization.

For protein analysis, six proteins (CD80, FOXP3, CD66b, PD-L1, PD-L2, and CD95/Fas) were excluded owing to failure to reaching the detection threshold in all ROIs. A signal-to-noise ratio (SNR) was obtained by dividing the counts of each antibody by the three IgG-negative control antibodies. Protein counts were normalized using housekeeping proteins (histone H3 and ribosomal protein S6). Based on 1,000 accelerated bootstrap repetitions, uncertainty was calculated by bias correction and 95% confidence intervals. All analyses were performed with version 3.6.3 and RStudio 1.4.1103 by R.

### Immune-Infiltrating Cell Analyses

Immune-infiltrating cell analyses were performed using NanoString’s SpatialDecon tool to classify immune-infiltrating cells from the gene expression dataset of each ROI. Different from other variance-stable least squares deconvolution tools, SpatialDecon uses the constrained log-normal regression algorithm, which is more consistent with the long tail of the gene expression data. SafeTME, a predefined robust immune-infiltrating cell expression matrix, was used to analyze immune infiltration.

### Immunofluorescence Staining

Serial paraffin-embedded renal sections of patients with ANCA-GN were analyzed by immunofluorescence staining as previously reported (31). Briefly, sections were deparaffinized and antigen retrieval was performed in microwave-heated Tris-EDTA buffer, pH 9.0 (AWI0152a; Abiowell, China), for 20 min, followed by blocking with 10% goat serum in phosphate-buffered saline (PBS), and were then incubated overnight at 4°C with the following primary antibodies: CD206 (18704; Proteintech, Rosemont, IL, USA), iNOS (MAB9502; R&D System, Minneapolis, MN, USA), CD31 (BBA7; R&D System), and cleaved caspase-3 (9661; Cell Signaling Technology). Secondary antibodies conjugated with Alexa Fluor dye were obtained from Abcam. DAPI (P36941; Invitrogen, Carlsbad, CA, USA) was used as a nuclear counterstain. Images were obtained using Zeiss Axio Scope.A1 (Carl Zeiss Canada Ltd., Toronto, Canada).

### Periodic Acid–Schiff Staining

Mounted kidney sections after immunofluorescence staining were rinsed in 1× PBS to remove cover glass. Subsequently, periodic acid–Schiff (PAS) staining was performed according to the instructions in the PAS stain kit (G1008; Servicebio, Wuhan, China). Images were obtained using Zeiss Axio Scope.A1 (Carl Zeiss Canada Ltd.).

### Statistical Analysis

Data were presented as the mean ± SD. One-way ANOVA followed by Bonferroni correction was applied to the comparison of more than two groups. Correlation was determined with Pearson’s correlation analysis. Statistical analysis was performed as shown in the figure legends using GraphPad Prism v9 (GraphPad software, San Diego, CA, USA).

## Data Availability Statement

‘The datasets presented in this study can be found in online repositories. The names of the repository/repositories and accession number(s) can be found below: Gene Expression Omnibus, accession number: GSE192996, available at https://www.ncbi.nlm.nih.gov/geo/query/acc.cgi?acc=GSE192996.

## Ethics Statement

The studies involving human participants were reviewed and approved by the Clinical Research Ethics Committee of the Second Xiangya Hospital of Central South University. The patients/participants provided written informed consent to participate in this study.

## Author Contributions

LY, YL, TD, PS, HL, LS, FL, and AC designed and conducted the experiments and acquired and analyzed data. CW and XF conducted the renal biopsy and collected the clinical data of patients. XZ and SY evaluated the renal pathology. LY, YL, FL, KL, JH, and AC wrote the manuscript. All authors contributed to the article and approved the submitted version.

## Funding

AC was supported by grants from the National Natural Science Foundation of China (no. 81800637) and Hunan Natural Science Outstanding Youth Fund Projects (no. 2021JJ10075). YL was supported by the National Natural Science Foundation of China (no. 81570622). PS was supported by grants from the National Natural Science Foundation of China (no. 81800649).

## Conflict of Interest

The authors declare that the research was conducted in the absence of any commercial or financial relationships that could be construed as a potential conflict of interest.

## Publisher’s Note

All claims expressed in this article are solely those of the authors and do not necessarily represent those of their affiliated organizations, or those of the publisher, the editors and the reviewers. Any product that may be evaluated in this article, or claim that may be made by its manufacturer, is not guaranteed or endorsed by the publisher.

## Supplementary Material

Supplementary Table S1 The demographic and clinical features of patients.

Supplementary Table S2 The histopathological features of the patients with ANCA-associated glomerulonephritis.

Supplementary Table S3 DSP probes list for mRNA.

Supplementary Table S4 DSP probes list for proteins.

Supplementary Figure S1 The principle of DSP transcription and protein profiles. Oligo-labeled probes with photocleavable linker target complimentary sequences for RNA assay **(A)**, while Oligo-labeled antibodies target specific antigens for protein assay **(B)**, which were incubated with samples. After selection of region of interests (ROIs), UV light was applied to specifically cleave the photocleavable linker, which released the DSP barcodes in each individual ROI **(C)**. Then the DSP barcodes were collected individually into 96-plate-well from each ROI **(D)**. For RNA assay, the DSP barcodes which are responsible for specific recognition of each mRNA (26bp label with blue and rose red) were incubated with primer pairs to amplify the products by PCR and then were sequenced **(F)**. For protein assay, the DSP barcodes were reacted with NanoString’s probe R and probe U **(G, H)**.

Supplementary Figure S2 Quality control of ROIs for the RNA assay. **(A)** Counts distribution of ROIs’ housekeeping genes (HKs). The geometric means of housekeeping genes were calculated logarithmic (log2) and presented in the figure. **(B)** ROIs’ nuclei counts and surface area assessment. The GeoMxTM DSP requires certain nuclei counts and surface area of each ROI. The nuclei counts and surface areas of all the ROIs have met the limit. **(C)** Correlation among housekeeping Genes. There were 32 HK genes in this experiment, and 3 of HK genes were randomly selected to show the correlations. The results showed a high correlation among TMUB2, ARMH3 and TLK2. **(D)** Selection of normalization method. Normalization methods include Top 25%, which uses the top 25% genes as the benchmark for normalization, as well as HKs and Neg Probe methods. The relation between HK and Top 25% shows a highly strong correlation. In this study, Top 25% method was used for normalization.

Supplementary Figure S3 Quality control of ROIs for the protein assay. **(A)** Counts distribution of ROIs’ housekeeping proteins (HKs). The geometric means of housekeeping proteins were calculated logarithmic (log2) and presented in the figure. **(B)** ROIs’ nuclei counts and surface areas assessment. The nuclei counts and surface areas of this study were qualified for further analysis. **(C)** The quality control of the expression of each target protein relative to negative control. The targets whose calculated value were always lower than or close to the background value were excluded in the further analysis (highlighted in blue color). **(D)** The expression correlations among housekeeping proteins. The results showed a high expression correlation between Histone H3 and S6.
