# Supplementary material for: Digital Spatial Profiling of Individual Glomeruli From Patients With Anti-Neutrophil Cytoplasmic Autoantibody-Associated Glomerulonephritis
Source: Front Immunol. 2022 Mar 2;13:831253. doi: 10.3389/fimmu.2022.831253 (PMC8924137; doi:10.3389/fimmu.2022.831253)
Supplement: Supplementary Table S1 — The demographic and clinical features of patients. [file Table_1.pdf]

Table 1 The demographic and clinical features of patients

Variables	Patients					
	ANCA1	ANCA2	ANCA3	ANCA4	MCD1	MCD2
Gender	Male	Female	Male	Male	Male	Female
Age (years)	56	51	67	67	30	21
eGFR[a] (ml/min/1.73m^2)	18.65	11.98	43.3	7.94	100	114.9
ACR (mg/g)	1511.65	776.64	79.54	1513.99	186.11	–
24h urinary protein (mg/d)	4215.53	1559.36	132.64	–	876.02	11954.89
Anti-MPO (CU)	>739.8	688.5	258.7	133.10	<3.2	<3.2
Anti-GBM (CU)	–	–	–	158.90	–	–
C3 (g/L)	0.99[b]	0.75[c]	0.73[c]	0.63[c]	1.04[c]	1.26[c]
C4 (g/L)	0.34	0.33	0.19	0.16	0.25	0.19
CRP (mg/L)	24.4	14.5	24.9	46.6	2.13	1.53

ACR, albumin-to-creatinine ratio;

[a] eGFR estimation according to CKD-EPI formula;

[b] Reference value for C3 is 0.7-1.4 g/L and C4 is 0.1-0.4g/L;

[c] Reference value for C3 is 0.79-1.52 g/L and C4 is 0.16-0.38g/L;
