# Supplementary material for: Digital Spatial Profiling of Individual Glomeruli From Patients With Anti-Neutrophil Cytoplasmic Autoantibody-Associated Glomerulonephritis
Source: Front Immunol. 2022 Mar 2;13:831253. doi: 10.3389/fimmu.2022.831253 (PMC8924137; doi:10.3389/fimmu.2022.831253)
Supplement: Supplementary Table S2 — The histopathological features of the patients with ANCA-associated glomerulonephritis. [file Table_2.pdf]

Table 2 The histopathological features of the patients with ANCA-associated glomerulonephritis.

| | Patients | | | |
|---|---|---|---|---|
| | ANCA1 | ANCA2 | ANCA3 | ANCA4 |
| Class [a] | Crescentic | Crescentic | mixed | Crescentic |
| Total number of glomeruli [b] | 20 | 18 | 18 | 15 |
| Normal glomeruli [c] | 1 | 0 | 7 | 0 |
| Crescents (%, n) | 80% | 88.9% | 16.7% | 86.7% |
| Cellular | 15 | 11 | 3 | 13 |
| Fibrous | 1 | 5 | 0 | 0 |
| Global glomerulosclerosis | 1 | 2 | 1 | 2 |
| Synechiae | 1 | 0 | 6 | 0 |
| Extensive ischemic changes | 0 | 0 | 0 | 0 |
| Segmental glomerulosclerosis | 1 | 0 | 1 | 0 |
| Bowman's capsule rupture (%,n) | 65% (13) | 66% (12) | 27.8% (5) | 86.7% (13) |
| Complement deposition | C1q (-)<br>C3 (2+) | C1q (2+)<br>C3 (2+) | C1q (-)<br>C3 (-) | C1q (-)<br>C3 (2+) |

[a] Berden scheme: DOI: https://doi.org/10.1681/ASN.2010050477;

[b] Total number of glomeruli means the maximum number of glomeruli in one of the sections excluding incomplete glomeruli on the edge.

[c] Normal glomeruli exclusion criteria includes synechiae and extensive ischemic changes.
