# Supplementary figures and images for: Digital Spatial Profiling of Individual Glomeruli From Patients With Anti-Neutrophil Cytoplasmic Autoantibody-Associated Glomerulonephritis
Source: Front Immunol. 2022 Mar 2;13:831253. doi: 10.3389/fimmu.2022.831253 (PMC8924137; doi:10.3389/fimmu.2022.831253)

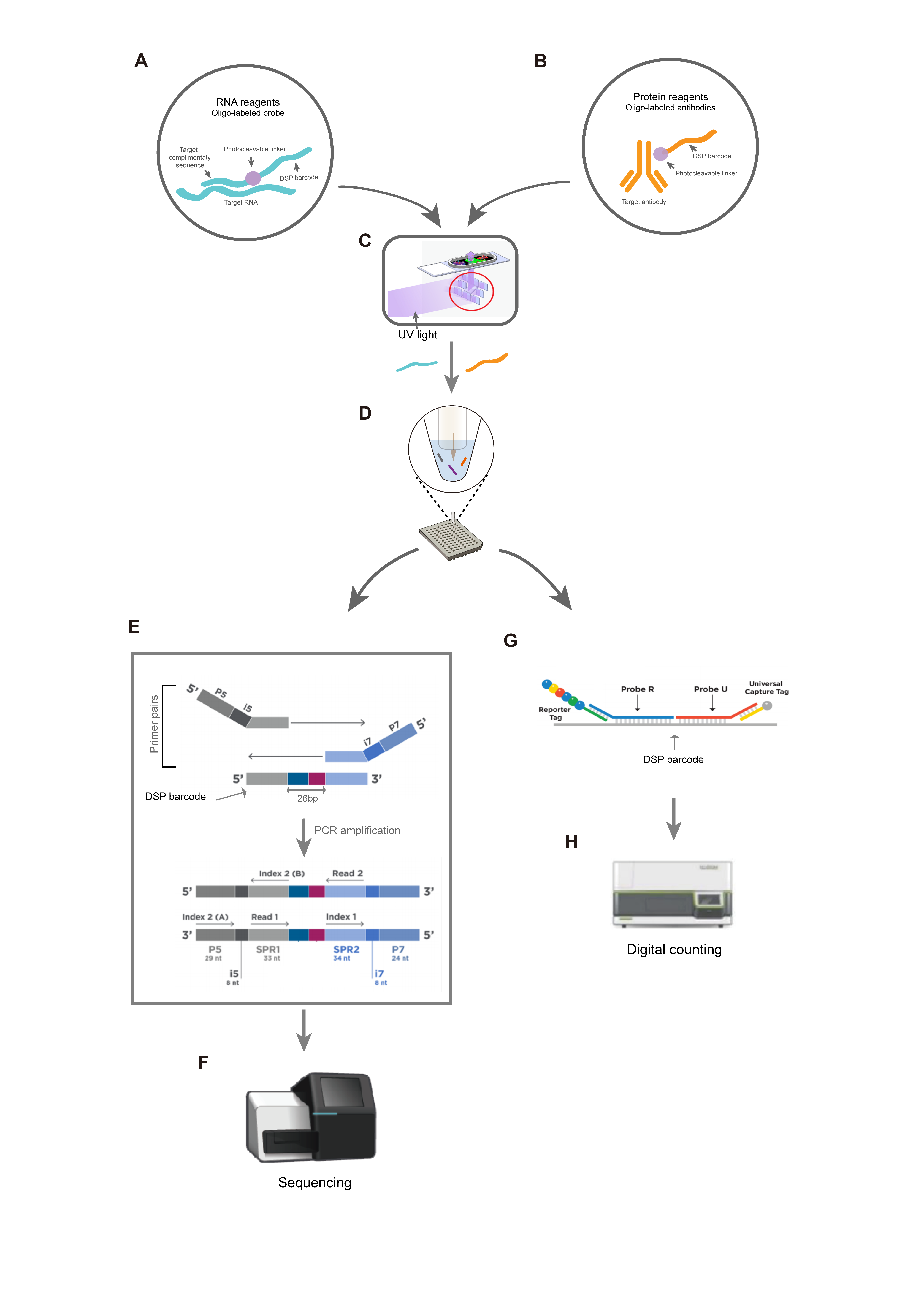

Supplement: Supplementary Figure S1 — The principle of DSP transcription and protein profiles. Oligo-labeled probes with photocleavable linker target complimentary sequences for RNA assay (A), while Oligo-labeled antibodies target specific antigens for protein assay (B), which were incubated with samples. After selection of region of interests (ROIs), UV light was applied to specifically cleave the photocleavable linker, which released the DSP barcodes in each individual ROI (C). Then the DSP barcodes were collected individually into 96-plate-well from each ROI (D). For RNA assay, the DSP barcodes which are responsible for specific recognition of each mRNA (26bp label with blue and rose red) were incubated with primer pairs to amplify the products by PCR and then were sequenced (F). For protein assay, the DSP barcodes were reacted with NanoString’s probe R and probe U (G, H). [file Image_1.tif]

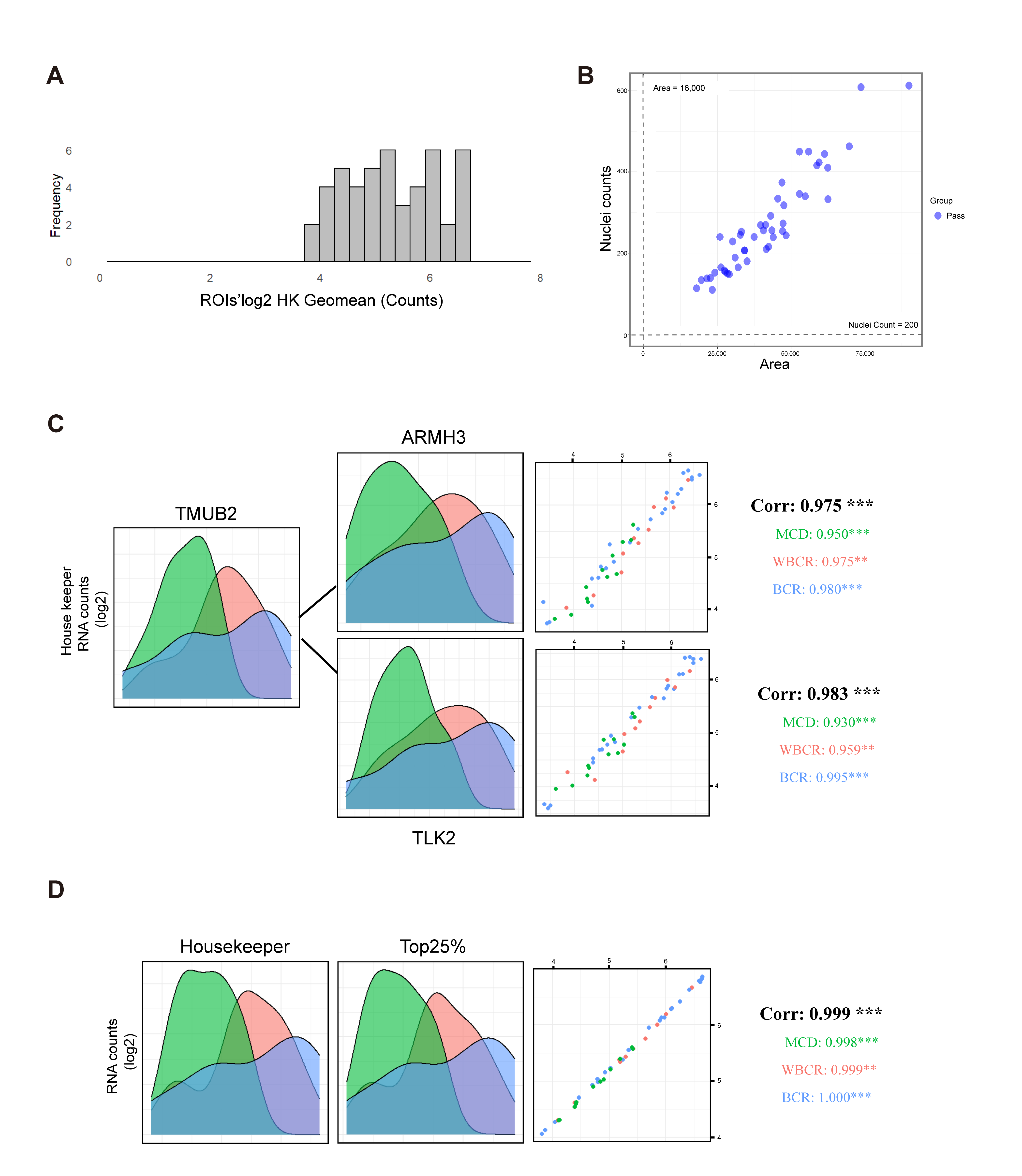

Supplement: Supplementary Figure S2 — Quality control of ROIs for the RNA assay. (A) Counts distribution of ROIs’ housekeeping genes (HKs). The geometric means of housekeeping genes were calculated logarithmic (log2) and presented in the figure. (B) ROIs’ nuclei counts and surface area assessment. The GeoMxTM DSP requires certain nuclei counts and surface area of each ROI. The nuclei counts and surface areas of all the ROIs have met the limit. (C) Correlation among housekeeping Genes. There were 32 HK genes in this experiment, and 3 of HK genes were randomly selected to show the correlations. The results showed a high correlation among TMUB2, ARMH3 and TLK2. (D) Selection of normalization method. Normalization methods include Top 25%, which uses the top 25% genes as the benchmark for normalization, as well as HKs and Neg Probe methods. The relation between HK and Top 25% shows a highly strong correlation. In this study, Top 25% method was used for normalization. [file Image_2.tif]

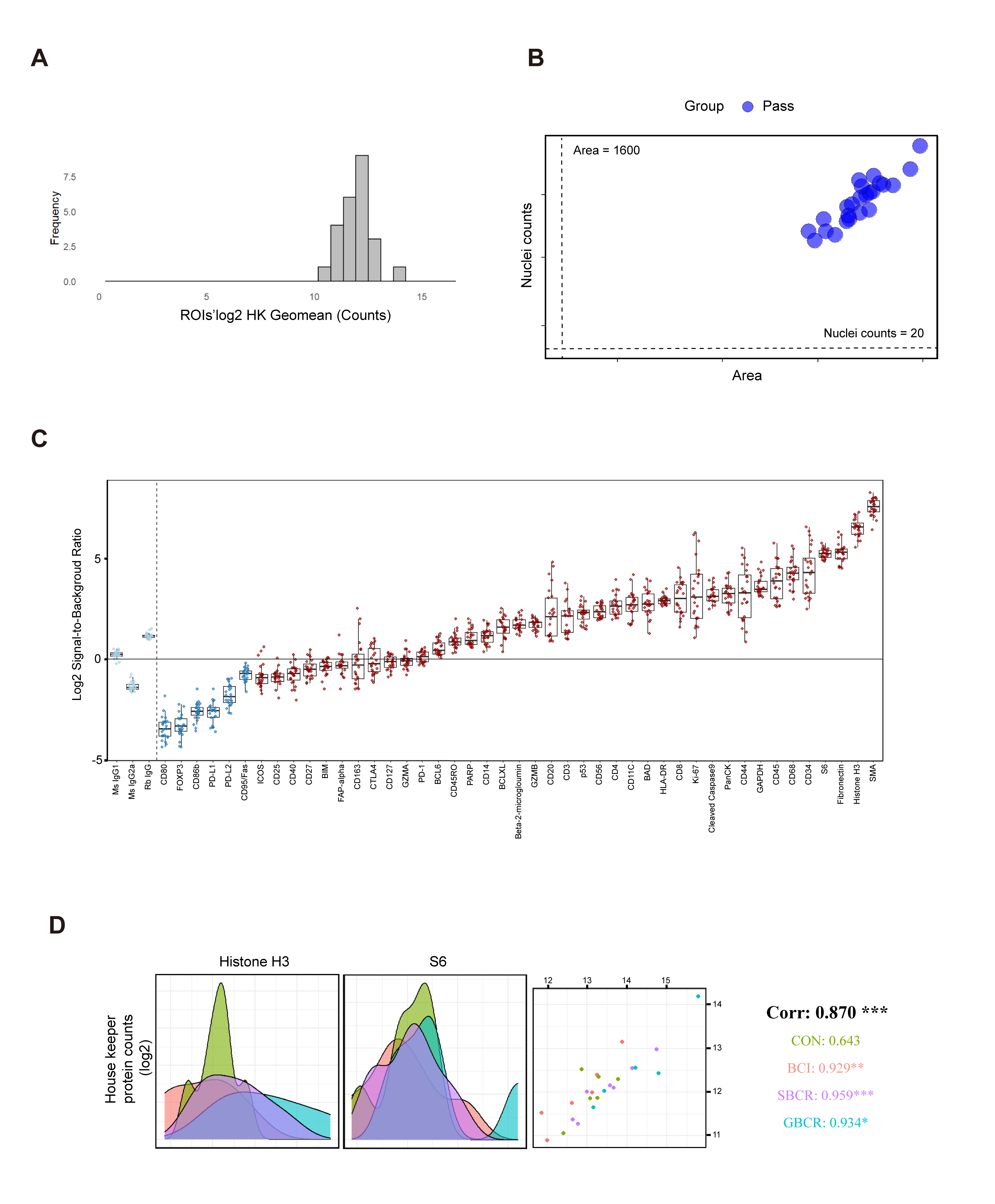

Supplement: Supplementary Figure S3 — Quality control of ROIs for the protein assay. (A) Counts distribution of ROIs’ housekeeping proteins (HKs). The geometric means of housekeeping proteins were calculated logarithmic (log2) and presented in the figure. (B) ROIs’ nuclei counts and surface areas assessment. The nuclei counts and surface areas of this study were qualified for further analysis. (C) The quality control of the expression of each target protein relative to negative control. The targets whose calculated value were always lower than or close to the background value were excluded in the further analysis (highlighted in blue color). (D) The expression correlations among housekeeping proteins. The results showed a high expression correlation between Histone H3 and S6. [file Image_3.tif]
